# Ethnicity and Population Structure in Personal Naming Networks

**DOI:** 10.1371/journal.pone.0022943

**Published:** 2011-09-06

**Authors:** Pablo Mateos, Paul A. Longley, David O'Sullivan

**Affiliations:** 1 Department of Geography University College London, London, United Kingdom; 2 School of Environment, University of Auckland, Auckland, New Zealand; University of Utah, United States of America

## Abstract

Personal naming practices exist in all human groups and are far from random. Rather, they continue to reflect social norms and ethno-cultural customs that have developed over generations. As a consequence, contemporary name frequency distributions retain distinct geographic, social and ethno-cultural patterning that can be exploited to understand population structure in human biology, public health and social science. Previous attempts to detect and delineate such structure in large populations have entailed extensive empirical analysis of naming conventions in different parts of the world without seeking any general or automated methods of population classification by ethno-cultural origin. Here we show how ‘naming networks’, constructed from forename-surname pairs of a large sample of the contemporary human population in 17 countries, provide a valuable representation of cultural, ethnic and linguistic population structure around the world. This innovative approach enriches and adds value to automated population classification through conventional national data sources such as telephone directories and electoral registers. The method identifies clear social and ethno-cultural clusters in such naming networks that extend far beyond the geographic areas in which particular names originated, and that are preserved even after international migration. Moreover, one of the most striking findings of this approach is that these clusters simply ‘emerge’ from the aggregation of millions of individual decisions on parental naming practices for their children, without any prior knowledge introduced by the researcher. Our probabilistic approach to community assignment, both at city level as well as at a global scale, helps to reveal the degree of isolation, integration or overlap between human populations in our rapidly globalising world. As such, this work has important implications for research in population genetics, public health, and social science adding new understandings of migration, identity, integration and social interaction across the world.

## Introduction

In recent years there has been an explosion of interest in analysing complex social phenomena through network representation [1]. A fundamental preoccupation in these approaches is to detect and understand the structure of social relationships, with a view to discovering or corroborating the observed behaviour of social groups [2]. One such phenomenon is the community structure of social networks, represented by densely interconnected clusters of nodes with relatively sparse external linkage [3]. The expectation is that the structure of such communities should clearly reflect patterns of social interactions in the real world, for example reflecting geographic, ethnic, religious, linguistic, gender or social class preferences, or constraints upon how we relate to one other. However, traditional algorithms to detect network community structure have struggled to cope with the extremely large networks derived from the recent availability of millions or even billions of digitized interactions between individuals, especially over the Internet [2], [3], [4]. New optimised algorithms for such very large networks have only very recently been proposed. This has in turn resulted in initial explorations of the network structure of complete national populations through interactions between individuals that are automatically collected from transactional data [4], [5], [6], [7], [8]. For example, researchers have automatically classified the 2.5 million users of a mobile phone operator in Belgium into French and Flemish speaking communities based exclusively on the topological network structure of their 800 million phone calls and texts interactions [9]. In doing so they have demonstrated the enduring importance of linguistic and geographical barriers in the age of global mobile communications, and more importantly, that they can automatically be detected using network analysis. Despite these advances two key obstacles remain, namely a) data availability issues, such as lack of public access to transactional datasets representative of complete populations, and b) methodological issues, such as devising appropriate network weighting metrics in order to highlight the most relevant links while removing the noise generated in extremely highly dense networks.

The motivation for our own research is to propose an automated method to detect the ethno-cultural relationships between people in large populations, using a readily available and underused resource. Our data derive from nationally representative electoral registers or telephone directories that make it possible to propose new network representations of complete populations' ethno-cultural structure as ‘naming-networks’. These are constructed from forename-surname pairings observed in the populations of 17 countries. Pairings are weighted according to new measures of naming proximity that are based upon the unequal probability of connectedness between names.

Naming practices are far from random, instead reflecting social norms and cultural customs [10]. They exist in all human groups [11] and follow distinct geographical and ethno-cultural patterns, even in today's globalised world. Any personal naming system serves two primary functions: to differentiate individuals from each other, and, simultaneously, to assign them to categories within a social matrix [11]. Names thus provide important information about social structure [12]. As such, “naming systems both reflect and help to create the conceptions of personal identity that are perpetuated within any society” [11] (page 167). The outcome is that distinctive naming practices in cultural and ethnic groups are persistent often even long after immigration to different social contexts [13], [14]. We exploit such regularities in this international investigation.

Our analysis utilizes the pairings of *surnames* (family names or last names), which normally correspond to the components of a person's name inherited from his or her family [10], and *forenames* (first names, given names, or Christian names), which refer to the proper name given to a person, usually at birth. Our work necessarily only applies to societies that use both types of personal names. The hereditary character and group identity function of *surnames* renders them useful to classify populations in demography [15], health [16] and genetics research [17], [18], [19], [20], [21], since they document ancestral proximity within and between populations and provide indicators of population structure [19], migration events [17], intermarriage [22], endogamy and genetic inheritance [20], [23]. More generally, research has identified the potential usefulness of surnames to classify health and population registers according to ethno-cultural origin of sub-populations [15], and even social on-line communities such as MySpace and Facebook [24] or Wikipedia [25]. In surprising isolation from surname research, the cultural distinctiveness in *fore-naming* practices has attracted wide and interdisciplinary attention in sociology [12], [26], geography [27], psychology [28], economics [14] and linguistics [29], [30] over recent decades. Such interest derives from the fact that parental selection of forenames is far from random since it arises out of the culture that a person is born into [29], alongside gender, class, ethnicity, religious affiliation, language and (post migration) identification with the host society [12]. The outcome is that distinctive naming practices in cultural and ethnic groups are persistent often even long after immigration to different social contexts [13], [14]. Although widely exposed, such regularities in sur- and fore- naming practices have been largely exploited in isolation from each other. Here, for the first time, we undertake extensive international analysis of the *combined* effects of forenames and surnames as indicators of cultural or ethnic ties in studies of population structure using a network analysis approach. This has not hitherto received systematic focus at the international level, although there have been seminal studies of naming practices in some individual countries by Tucker and Hanks [10], [13], [31], [32]. These use forename-surname pair frequencies to classify surnames in a probabilistic way, but only studied first order relationships (a name and its immediate neighbours) and not their overall network topologies.

Our contribution is to conceptualise the ethno-cultural relationships between people as a network representation of personal names (vertices or nodes) connected by weighted forename-surnames pairs (links or edges). Such networks are derived from complete population registers such as telephone directories or electoral registers. Here, our main empirical analysis entails unsupervised classification of the topological structure of a naming network to detect ethno-cultural clusters using population registers from 17 countries across three continents. Surname networks are then extracted from the full network and weighted using relative frequencies of occurrence of shared forenames. We demonstrate that they have distinctive structure, which can be related to cultural, ethnic, and linguistic groups, and that they can reveal details of socio-cultural structure that are hard to identify by other methods. Our hypothesis is that the structure of such networks mirrors socio-cultural structures in populations. Drawing a parallel with *amazon.com*'s recommendation service; “people who bought this book also bought…” we could say that “people who bear this surname often choose these forenames”. Pursuing this analogy, just like book titles at *amazon.com* have automatically been clustered into genres using purchasing behaviour in a network representation [33] we propose to cluster surnames into cultural, ethnic and linguistic groups of forenaming preference in a similar fashion using population registers. As such, to our knowledge this is the first study to propose and test this type of empirical approach to detect the ethnicity structure of whole populations using people's names.

## Methods

### Building naming networks

**Figure 1:**
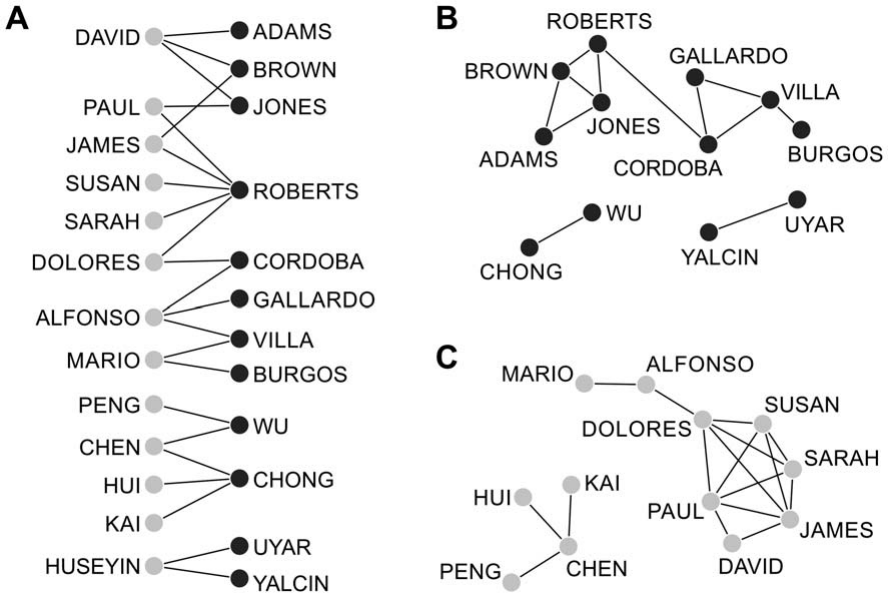
Simple naming networks derived from a population of twenty-three people. Figure 1A shows a two-mode network of 23 people, comprised of 13 unique forenames (blue nodes) and 12 unique surnames (red nodes) connected by 23 links each representing one person. Figures 1B and 1C are one-mode transformations from network 1A. Figure 1B shows a one-mode network of the 12 surnames linked by common forenames, while Figure 1C shows a one-mode network of 13 forenames linked by common surnames. Four CEL clusters emerge in 1B; Anglo-Saxon, Spanish, Chinese and Turkish. Notice that the first two CELs networks are joined together by a cross-CEL name (‘Dolores Roberts’).

The key idea underpinning the naming networks approach presented here is that cultural-ethnic-linguistic (hereinafter ‘CEL’) affiliations and practices are revealed as topological structures in a network in which unique forenames or surnames are considered as nodes, linked via common bearers. For any large population, network structure will manifest CEL communities [10] separated by the ‘social distance’ of distinctive naming practices [34]. Figure 1 presents an illustrative two-mode (bipartite) network based upon forename and surname (*fs*) associations of 23 people (Figure 1A), along with two derived one-mode associations based upon surnames (*ss*) (Figure 1B) and forenames (*ff*) (Figure 1C) alone. CEL cluster strength is reinforced by using one-mode networks, because of the multiplicative effect of combining the non-randomness of *fs* and *sf* links into a one mode (*ss* or *ff*) network. Here we will use only one-mode networks, defined by the preponderance of common cross-occurrences of (fore- or sur-) names within CEL communities, and their relative absence between communities.

Our fundamental premise is that the number of occurrences of a particular forename – surname pair *n_fs_* will substantially exceed a naïve expectation of its rate of occurrence were forenames randomly selected from a population. Thus
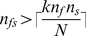
(1)where *k* is some rate (*k*≫1) by which we require the observed number of cases of the forename-surname pair *n_fs_* to exceed the naïve expectation, given *n_f_* occurrences of the forename and *n_s_* occurrences of the surname in the total population of *N* people. Observed name associations are retained if the observed frequency exceeds expectations by a threshold *k*. The threshold *k* may be considered a measure of the *naming unexpectedness* of a particular forename – surname combination within the pool of all names present in a society. Raising this threshold value focuses attention on the most strongly over-represented *fs* name-pair combinations, identifying the most tightly knit naming communities. The resulting threshold value applied to *n_fs_* is rounded up to the nearest integer count. This has the effect of removing from consideration name-pairs which occur only once (in practice a large number of pairs) which might otherwise be considered important because even one instance is many times more frequent than a naive (random) expectation would suggest.

### Weighting naming networks

An important consideration is how we assign weights to the *fs* links in the two-mode network. Rather than simply use the number of occurrences *n_fs_*.of each name-pair combination, because we are primarily interested in identifying surnames strongly linked to one another by shared forenames, we define an *fs* weight as:
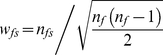
(2)The weight *w_fs_* reflects the importance to forename *f* of the *fs* link it shares with surname *s* (i.e. the number of people called “forename *f* – surname *s*” or *n_fs_* compared to the total frequency of forename *f* in a population). This approach is asymmetric in that if the aim was to cluster forenames strongly connected by shared surnames, it would be necessary to replace *n_f_* in the denominator of (2) with the frequency of occurrence of the linked surname (*n_s_*). A variety of formulations for *w_fs_* were investigated, and it was found that provided that the weights increase with *n_fs_* and decrease with the frequency of the forename in the population (*n_f_*), the final outcome is not much affected. This approach reduces the importance of very common names that bridge CEL clusters (weak ties) in the one-mode network, and is desirable because such ‘cosmopolitan’ names (e.g. ‘Maria Smith’ or ‘John Patel’) tend to obscure the distinctiveness of naming communities.

### Naming proximity

So far our analysis has dealt with a two-mode (bipartite) network, which can conveniently be represented as a sparse coincidence matrix (**W**) of *n_f_* rows by *n_s_* columns. In such a matrix, non-zero entries represent the existence of the forename-surname combination *fs* with their *w_fs_* weights value as per equation (2). However, we now need to transform this two-mode network into one mode graphs of either surnames or forenames as discussed above (Figures 1B and 1C). This produces square matrices of dimension *n_s_* by *n_s_* or *n_f_* by *n_f_*, respectively. We perform this transformation by matrix multiplication operations as follows:
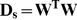
(3)

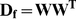
(4)where **D_s_** and **D_f_** are distance matrices of the one-mode surname and forename networks respectively. The final weight *w_ss_* between two surnames in matrix **D_s_** (their strength of connection) is given by the sum of products of the multiple *w_fs_* connections to their shared forenames (i.e. forenames shared between all bearers of both surnames). We describe this as the *naming proximity* (NP) between each pair of surnames *x* and *y*. Using equation (3), this can be expressed as
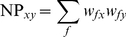
(5)Substituting (2) in (5) we formally define *naming proximity* (NP) between distinct surnames *x* and *y* as:
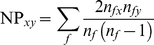
(6)where *x* and *y* are distinct surnames, summation is over all shared forenames *f*, *n_fx_* and *n_fy_* denote the frequency of occurrence of the forename-surname combinations *f−x* and *f−y* and *n_f_* is the overall frequency of occurrence of forename *f*. In this paper we cluster only surname networks linked via forenames, but the same procedure could in principle also be applied to forename networks.

### Data

One of the key strengths of the approach presented in this paper lies in the ease of access to population register data to build a global naming network, as well as the availability of published work on the CEL origins of many names. Our analysis consisted of two stages. First, we developed a preliminary clustering analysis of the ethnically diverse population of Auckland, New Zealand, to demonstrate the existence of population structure in naming networks without any prior knowledge of CEL groups. Second, we extended this network clustering analysis using a global synthetic network covering 17 countries in four continents, using a custom built dictionary of name origins to ascertain the CEL provenance of each cluster and to assess the accuracy of our automatic classification procedure.

Data used for this analysis derive from a very extensive database of 300 million people's names from 26 countries in four continents, assembled from publicly available telephone directories and electoral registers for a project developed at University College London (see worldnames.publicprofiler.org/). This database has been used, *inter alia* to build maps of population ethnic origins [35], [36], to measure residential segregation [37] and to classify populations in public health registers [38], [39] through a name classification known as *Onomap* (www.onomap.org).

The first subset extracted from the dataset is the 887,021 electors resident in the City of Auckland, New Zealand as recorded in the 2008 Electoral Register (hereinafter ‘Auckland dataset’). This subset comprised 79,855 unique surnames and 88,760 unique forenames, constituted in a two-mode network with 711,807 unique forename-surname pairs (links or edges).

The second subset of this database was created comprising records from 17 countries in Europe and the Indian subcontinent (see Table 1 for a full list of countries and name frequencies), in order to exclude imported naming systems in countries settled by colonisation – in which intermarriage between ancestral ethnic groups is likely to be greater. The extracted dataset comprised 118.3 million individuals in 17 countries, organised in a forename-surname network with 4.6 million unique surnames and 1.5 million unique forenames (hence 6.1 million nodes), and 46.3 million unique forename-surname pairs (links or edges: an average of 2.55 people per *f−s* pair).

**Table 1:** Description of the global names dataset with 17 *WorldNames* countries.

Country Name	Year	Country's Population	Individuals in WorldNames	Forename-Surname pairs
Austria	1997	8,316,487	2,516,864	1,707,653
Belgium	2007	10,511,382	3,378,147	2,504,949
Denmark	2006	5,457,415	3,075,509	1,153,183
Ex-Yugoslavia (*)	2006	10,159,046	1,704,633	757,355
France	2006	64,102,140	20,257,382	11,077,105
Great Britain	2006	60,587,300	45,688,172	11,454,381
Hungary	2006	10,064,000	281,305	162,683
India (4 city-regions **)	2004	*n/a*	321,662	250,818
Italy	2006	59,131,282	15,907,519	8,438,659
Luxemburg	2006	480,222	112,434	107,198
Norway	2006	4,770,000	3,581,614	2,071,687
Poland	2007	38,518,241	8,015,669	3,244,993
Romania (Bucharest)	2006	*n/a*	333,545	234,812
Slovenia	2007	2,019,245	344,709	277,934
Spain	2004	45,116,894	10,397,093	2,769,590
Sweden	2004	9,142,817	792,421	570,357
Switzerland	2006	7,508,700	1,559,532	1,204,039
**Total**			**118,268,209**	**47,987,396**

Summary of key characteristics from the global names dataset from 17 countries extracted from W*orldNames*. The year refers to the publication date of the telephone directory (Electoral Register in Great Britain), and the country's population refer to the closest available year. (*) Ex-Yugoslavia in 2006 includes current day Serbia, Montenegro & Kosovo; (**) the four city-regions in India are Delhi, Mumbai, Chennai, Hyderabad metropolitan areas.

Additionally, a reference list of ‘diagnostic’ surnames whose cultural provenance is known was compiled from the academic literature and official statistical sources, in order to validate the results of network clustering. This reference list was compiled by manually searching for published sources with lists of surnames and their linguistic, ethno-cultural or geographical origin. In our inclusion criteria we deliberately discarded the use of surname dictionaries (to avoid possible copyright issues), only included sources that used surname frequencies (used in order to exclude rare names and give a greater level of validity to the CEL assignment) and only used information derived from peer-reviewed publications or national statistics websites that report surname frequency per nationality or country of birth. 25 different data sources were used and are listed in Text S1. Three additional sources were neither peer-reviewed nor part of national statistics, but were used in order to include some missing CELs that would otherwise not have been covered: they nevertheless came from trusted authors and institutions. The reference list comprised 30,479 surnames, each identified with one of 40 cultural ethnic and linguistic groups (CELs) coded following Hanks and Tucker's typology [32] (see Tables 1 and 2 for full details). The reference list of diagnostic surnames used in this paper was taken to be the ‘gold standard’ against which the accuracy of the automatic network clustering method could be evaluated.

**Table 2:** List of CEL groups and name frequencies extracted from the global dataset.

CEL code	CEL name	Number of unique surnames	Number of forename-surname pairs	Total number of people
afg	Afghan	255	1,525	1,907
afr	African	73	16,788	37,089
ara	Arabic	2,747	62,181	134,183
arm	Armenian	25	76	90
bri	British	80	308,143	8,455,394
bul	Bulgarian	17	2,428	4,057
cam	Cambodian	67	3,514	4,305
chi	Chinese	974	171,843	346,654
czk	Czech & Slovak	88	17,941	31,948
dan	Danish	20	78,877	1,558,343
dut	Dutch	115	90,344	335,331
fin	Finnish	10	1,596	5,899
fre	French	149	200,825	2,021,921
ger	German	62	98,722	489,983
gre	Greek	223	9,719	21,001
hun	Hungarian	92	38,521	137,040
ind	Indian	901	139,698	376,322
iri	Irish	26	42,422	682,850
ita	Italian	147	250,527	1,445,061
jap	Japanese	1,851	16,917	19,808
jew	Jewish	35	18,342	45,682
kor	Korean	82	3,990	5,623
lit	Lithuanian	20	241	261
nig	Nigerian	14	1,496	1,971
nor	Norwegian	83	112,860	759,288
pak	Pakistani	597	79,395	241,847
per	Persian	4,775	34,744	39,123
pol	Polish	196	87,723	1,202,623
por	Portuguese	20	35,478	162,787
rom	Romanian	37	11,717	27,862
rus	Russian	17	306	381
sla	Slavic	9	5,212	18,440
slo	Slovenian	96	26,493	53,440
spa	Spanish	880	667,778	5,477,346
ssl	South Slavic	199	118,456	776,359
sud	Sudanese	135	616	653
swe	Swedish	18	67,752	219,181
tur	Turkish	2,174	67,049	92,013
ukr	Ukrainian	18	1,087	6,221
vie	Vietnamese	84	16,397	38,078
**Total**		**17,411**	**2,909,739**	**25,278,365**

CEL = Cultural Ethnic and Linguistic groups. Definition of CELs and abbreviations adapted from Hanks and Tucker [31], [32]. Out of the 30,479 unique surnames collected in the reference list (see text under “*Methods*; *Data*” section) only 17,411 were present in the global names dataset (17 countries selected from *WorldNames*). This table lists the surname frequency distribution per CEL and the number of forename-surname pairs in which they are involved in the global names dataset used in this paper.

### Network clustering analysis

The two datasets used in this analysis (Auckland's and the global 17-country), are simply large registers of people's names, listing each person's forename and surname. These raw records were aggregated into forename-surname pairs along with their frequencies. They were initially represented as a two-mode (bipartite) network of forenames and surnames as nodes linked by forename-surname pairs as edges in a similar fashion to Figure 1A. This two-mode network was subsequently transformed into a one-mode surname-to-surname (*s-s*) network and the unexpectedness rate (*k*) and naming proximity (*NP*) weights calculated for all links as specified in the previous section.

After finalisation of each weighted *s-s* one-mode network, standard network clustering algorithms were applied to detect its community structure [3]. We have tested three different algorithms to find communities in very large networks following the criteria that they are able to handle very large weighted networks (up to ten thousand nodes and around a million edges) and that the chosen algorithm be implemented in some form of software capable of running within hours using a powerful desktop computer. The three candidate algorithms were *Fastcommunity* [4], *Walktrap* [7] and *Label propagation* [8] which were all tested for their suitability in finding communities in very large naming networks. Clustering performance was measured using *modularity* (*Q*), defined as the quotient of the number of edges that fall within clusters to the number outside the clusters [3]. *Walktrap* and *Label propagation* repeatedly came up with identical results, which were always outperformed by *Fastcommunity* in terms of higher modularity (*Q*) values. For ease of interpretation and conciseness the main paper only reports results based on the *Fastcommunity* clustering algorithm.

## Results

### Auckland's naming network

**Figure 2:**
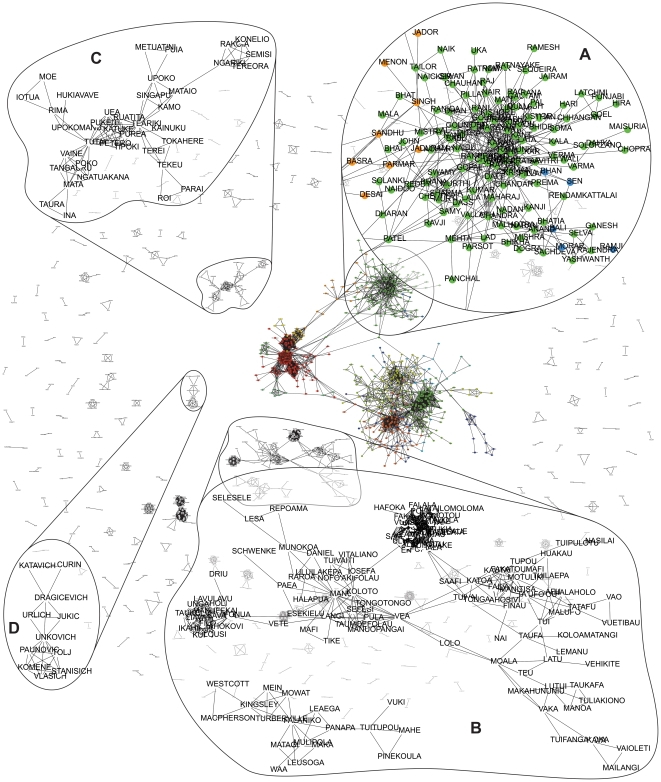
Naming network of the city of Auckland New Zealand. The Auckland surnames network filtered at *k*>100, *NP*> = 0.0. The graph shows the highly structured outcome of naming practices in a city with high rates of immigration from all over the world. The giant component in the centre of the graph has been classified with *fastcommunity* algorithm into 22 clusters, each depicted by a different node colour. Four subgraphs are magnified to show the tightly knit internal structure of some CEL communities. One (fig. 2A) is classified as part of the giant component (and is South Asian/Indian), the others are Tongan (fig. 2B), Samoan and other Pacific Islanders (fig. 2C), and Eastern European (particularly Dalmatian: fig. 2D). The last three are disconnected from the network giant component.

The case study of Auckland, New Zealand, was chosen as a good example of a small yet ethnically diverse population of a single city, which has hitherto received very little attention in the naming literature. The naming network of Auckland's 887,021 registered electors is shown in Figure 2 transformed into a surnames network and filtered at *k*>100, *NP*> = 0.0 (i.e. no *NP* filtering). We believe that this is the first naming network ever drawn of a complete city's population. The graph shows the highly structured outcome of naming practices in a city with high rates of immigration from all over the world, in which tightly knit clusters are strongly suggestive of CEL communities. In the centre of the graph, one giant connected component reflects the ‘majority of the population’ whose surnames are connected with the largest number of other surnames through shared forenames. Visually, we can easily distinguish three distinct sub-components within this giant component, but its structure becomes much clearer after applying a community detection algorithm. Such network clustering techniques necessarily only work on a single connected component in a network, since the presence of any other isolated components already reflects membership of different communities (i.e. no clustering required). Therefore, we applied the *fastcommunity* algorithm to the giant component at the centre of Figure 2. We classified all of the surnames into 22 clusters, depicted using different colours in the graph. One of the three sub-components is magnified in order to expose its surnames and structure (fig. 2A), in this case names of South Asian origin, with the three node colours assigned by the cluster analysis indicating likely internal sub-structure (orange denotes Sikh, and green and blue different regions of India). We have noticed that this giant component includes the most common names that are also the most likely to be found in other countries and also in the literature that traces each name's ethno-linguistic origins. However, if we turn our focus to the rest of the components in the graph, disconnected from the giant component, we find very interesting unique CEL communities that are particular to New Zealand. Three of these smaller components are magnified to show the tightly knit internal structure of their CEL communities, which from local knowledge we know are; Tongan (fig. 2B), Samoan and other Pacific Islanders (fig. 2C), and Eastern European (particularly Dalmatian, a late 19^th^ century immigrant group: fig. 2D). Other much smaller components are scattered around the periphery of this ‘constellation of naming galaxies’. These can be visualized in an on-line version of Figure 2 available at http://www.onomap.org/naming-networks/fig2.aspx: this Figure can be navigated with full panning and zooming capabilities for flexible exploration. The obvious tightly knit and geometrically compact topologies clearly show the outcome of the exclusive nature of naming practices, as predicted by the literature reviewed above. It is striking that such clear ethno-cultural structure within a single city automatically emerges from the naming network representation proposed here, even without previous knowledge on the origins of these names or the existence of such communities in Auckland.

Some additional findings and implications of this initial network analysis should be mentioned here. The application of threshold values of *k* and NP to the raw data reduces the number of nodes and links in the network, through filtering out large numbers of common names that are not distinctive of particular naming communities. The example shown in Figure 2, with no NP filtering and *k*>100, filters out a large number of Anglo-Saxon names in Auckland that are of English, Scottish, Welsh, Cornish or Irish extraction. Use of a lower *k* filter threshold leads to retention of more of these common names, but the communities that are detected through automatic clustering are less distinctive. Furthermore, as previously discussed, network clustering algorithms work only on fully connected components of the network. Therefore, in order to complete the CEL detection methodology, the clustering algorithm would need to be repeated for all of the other components of Figure 2. Finally, it is important to note that each component in the network does not necessarily correspond to a single CEL (since the giant one does not), and in fact the smaller ones on the sides could even represent individual families, or small sub-communities, several of which would need to be joined-up in order to form a single CEL group. Based on the interesting and interpretable structure identified in the Auckland data, we believe that further development of our approach will enable us to retrieve additional structure, including the more common communities and names associated with them. We explore the potential of the method further in the global names analysis.

### Global naming network

**Figure 3:**
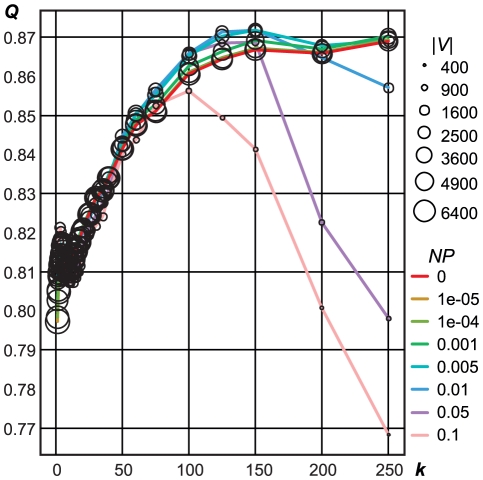
Modularity results for different values of *k* and *NP* thresholds. Each point (circle) in the graph shows the modularity results (Q, *y*-axis) of running the *fastcommunity* algorithm [4] on one-mode surname networks filtered using different values of *k* (*x*-axis), and naming proximity (*NP* as line colours), with the sizes of the circles (|V|) depicting the number of surnames (nodes) in the filtered network.

After demonstrating the existence of such clear structure in naming networks for a single city, we proceeded to undertake an analysis of the much larger 17 country ‘global dataset’. The diagnostic list of 30,479 surnames for which origins are asserted in published sources (see Text S1) were linked to the matching surnames in the extracted global dataset (see Tables 1 and 2). The resulting two-mode network had 17,411 surnames linked to 243,135 forenames through 2,909,739 unique forename-surname pairs, and their breakdown by CEL group is listed in Table 2. We experimented with threshold values of *k* (equation 1) and *NP* (equation 6) when transforming this two-mode network into a one-mode surname network measuring the performance of *fastcommunity* in terms of modularity values (Q) and the final number of surnames (nodes(|V|) in the filtered network. Some results of this experimentation are shown in Figure 3 and demonstrate that over-representation of a forename with respect to a surname (*k*) drives the success of the clustering results, rather than the naming proximity metric (*NP*).

After filtering this global surname network at *k*> = 150 and *NP*> = 0, a giant component comprised of 5,787 nodes (surnames) was classified into 82 clusters using *fastcommunity*. The breakdown of surnames in each of the largest 20 clusters belonging to each CEL in the reference list is summarised in Table 3. For example cluster 4 is 86% Chinese while cluster 9 is 68% Greek and cluster 13 is 98% Japanese. The great majority of these surnames (77%) were assigned to clusters with a single CEL allocation in the reference list. The remainder presented a mix of multi-origin names or culturally close CEL groups, such as different Romance, Slavic, Germanic or Nordic languages, or Muslim names that cannot be attributed to a single CEL group. To accommodate some of these overlaps, pairs or triads of the largest 20 clusters were amalgamated into 14 clusters if they contained the same CEL or culturally similar CELs (see Tables 3 and 4). Addition of these clusters increased the percentage of surnames ‘correctly’ classified to 85%. Measures of binary classification success were calculated for the 14 amalgamated clusters, with very satisfactory results as shown in Table 4 (Sensitivity: 0.71–1; Specificity: 0.96–1; Positive Predictive Value: 0.52–1; Negative Predictive Value: 0.96–1; with ranges denoting extreme values for different CEL groups).

**Table 3:** Validation of clustering results: Percentage of surnames in cluster by reference CEL group.

CEL group (ref. list)	Cluster ID (largest 20)																				Nr. of surnames
	1	2	3	4	5	6	7	8	9	10	11	12	13	14	15	16	17	18	19	20	
**afr**	**52**																				55
**ara**	13	**75**			1				1		3	3			9	7				1	469
**bri**			**73**																		13
**iri**			20											1							5
**chi**	10	2		**86**	40		5	7		3	2	1	1		1		3	10		1	466
**vie**				1	32													2			44
**dut**					2	**100**	**75**	**53**													46
**fre**	3		7		20		20	40						1			2	7			63
**gre**									**68**												58
**ind**	4	2		1							**83**	**87**			8	1		2			541
**jap**	2			2							1	1	**98**	1	1		3				343
**nor**														**55**							64
**dan**														17							20
**swe**														15							17
**pak**	2	2			1						3	4			**50**	1					291
**per**	6	17		1	1				4	4	4	3		1	24	**89**				2	778
**spa**	3			1	1				1					2			**72**	44			629
**por**																		32			20
**ssl**									4	6	1			1					**100**		103
**tur**		1							4	2		1			2	1				**93**	1042
**czk**									1	16											46
**slo**				1						24											66
**hun**										30											78
**Other CELs**	5	1	0	7	2	0	0	0	17	15	3	0	1	6	5	1	20	3	0	3	**297**
**Grand Total**	100	100	100	100	100	100	100	100	100	100	100	100	100	100	100	100	100	100	100	100	5554
**Nr. of unique surnames**	104	444	15	384	126	20	20	15	78	249	371	189	302	117	507	573	814	59	80	1087	5554
**Most probable cluster-CEL**	afr	ara	bri	chi	chi-vie	dut	dut	dut-fre	gre	hun-slo-czk	ind	ind	jap	nor-dan-swe	pak-per	per	spa	spa-por	ssl	tur	

The table shows clustering results on the global network filtered at *k* = 150 and NP = 0. The columns represent the largest 20 clusters and the rows the CEL groups in the diagnostic list, while the rows are a selection of 23 CELs with higher values in the table. The cell values are the percentages of unique surnames within each cluster that matches a particular CEL group in the reference list ( = >50 highlighted in bold). Percentages are rounded to the nearest integer and zero values are not shown. The largest 20 clusters shown here account for 5,554 surnames out of a total of 5,787 surnames assigned to 82 clusters. The last row lists the most probable CEL allocation (or CEL combination) to each cluster based on the highest percentages. For example, cluster 4 is 86% Chinese, while cluster 9 is 68% Greek, while cluster 6 is 100% Dutch (see table 2 for a description of CEL codes).

**Table 4:** Binary classification results of 14 families of CEL groups.

Families of CEL groups	afr	ara	bri	chi-vie	dut	gre	hun-slo-czk	ind	jap	nor-dan-swe	pak-per	spa-por-ita	ssl	tur
**Amalgamated Cluster ID (** **table 3** **)**	1	2	3	4; 5	6;7;8	9	10	11;12	13	14	15;16	17;18	19	20
**Nr. of Surnames**	104	444	15	510	55	78	249	560	302	117	1080	873	80	1087
**True Positives**	54	333	11	422	43	53	173	473	295	101	890	769	80	1013
**False Positives**	50	111	4	88	12	25	76	87	7	16	190	104	0	74
**False Negatives**	1	136	2	88	3	5	17	68	48	0	179	19	23	29
**True Negatives**	5449	4974	5537	4956	5496	5471	5288	4926	5204	5437	4295	4662	5451	4438
**Classification accuracy**														
**Sensitivity**	0.98	0.71	0.85	0.83	0.93	0.91	0.91	0.87	0.86	1.00	0.83	0.98	0.78	0.97
**Specificity**	0.99	0.98	1.00	0.98	1.00	1.00	0.99	0.98	1.00	1.00	0.96	0.98	1.00	0.98
**PPV**	0.52	0.75	0.73	0.83	0.78	0.68	0.69	0.84	0.98	0.86	0.82	0.88	1.00	0.93
**NPV**	1.00	0.97	1.00	0.98	1.00	1.00	1.00	0.99	0.99	1.00	0.96	1.00	1.00	0.99

This table summarises the binary classification results of an amalgamation of the 20 clusters shown in Table 3 into 14 amalgamated clusters that correspond to CEL families of one, two or three closely related CEL groups (as specified in the second row). The top half of the table shows the raw counts of surnames correctly or incorrectly classified according to the reference list, while the bottom half reports results of measures of classification accuracy (Sensitivity, Specificity, PPV = Positive Predictive Value, NPV = Negative Predictive Value).

**Figure 4:**
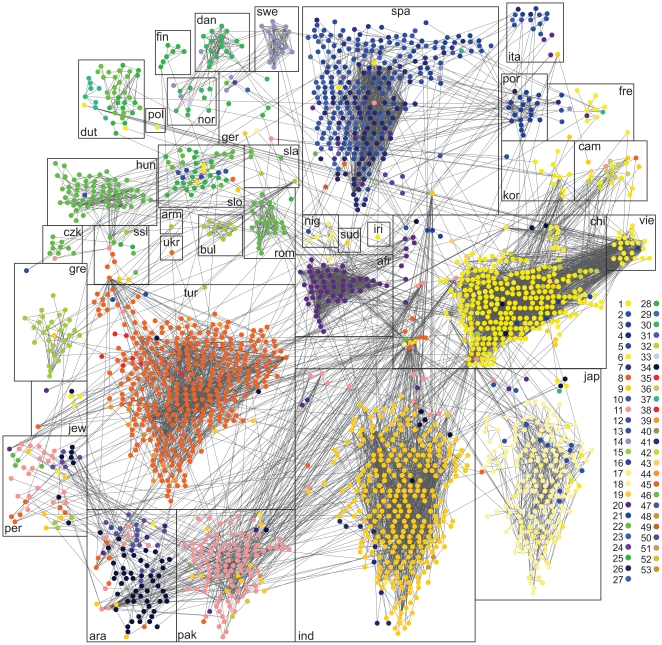
Cultural clusters in the global surname network. Global surname network from 17 countries with 2,232 nodes (surnames) and 7,515 edges (shared forenames between each surname pair). Each node is coloured according to the cluster assigned by *Fastcommunity* (*k*>150 *NP*> = 0.01 producing 53 clusters), while the rectangles group surnames assigned to the same CEL group in the reference list (see Table 2 for CEL abbreviations).

In order to produce a graph that is less dense and that can be clearly visualised, the global surname network was filtered using values of *k*> = 150 and *NP*> = 0.01, as shown in Figure 4 (navigable version at http://www.onomap.org/naming-networks/fig4.aspx). The network's giant component comprised 2,232 surnames and was classified using *Fastcommunity* into 53 distinct clusters (node colours in Figure 4). Cluster assignments remained consistent with those from the CEL reference list (shown with bounding boxes). The layout of sub-clusters within the graph, which places nodes in proximity to their directly connected nodes, clearly shows a geographical proximity arrangement of CELs. This layout is an emergent property of the network data (i.e. its link topology and weights), and it can be argued that it parallels other maps of relatedness between populations extracted from genetic data [40]. There are frequent overlaps between some culturally close groups (e.g. between Spanish, Italian and Portuguese or between Chinese, Vietnamese, Cambodian and Korean names). CELs that are proximal in ethno-religious space, rather than in a geographical sense, also appear to share naming practices (e.g. Turkish, Arab, Persian and Pakistani names), or those close geographically but distant in ethno-religious space are distinctly clustered yet separated (e.g. Indian and Pakistani names or Chinese and Japanese names). Furthermore, it is striking to notice that although the global data are drawn principally from European countries, it is non-European CEL groups which show up clearly in the network analysis community structure. As we have argued, this is again proof of the distinctiveness of naming practices that are preserved after migration.

Methodologically, in order to produce Figure 4 we applied an additional low threshold filter of NP> = 0.01, in order to reduce the number of surnames (nodes, from 5,787 to 2,232) and *s-s* links in the network. This retains only the most tightly connected names in the analysis, and leads to the formation of a reduced number of clusters (53 instead of 82). In fact, this combination of the *k* and NP filters results in the removal of all surnames in the dataset that belong to four CELs (*afg*, *bri*, *lit*, *rus*), and hence they are not present in Figure 4. This arises either because of the small number of surnames in the reference list in some of these CELs (*rus*, *lit*: see Table 2) or because they are very common surnames, and hence more prone to present high *f-s* connectivity with other CELs (*brit*), and hence are eliminated by the filters applied. With respect to the former issue it is worth noting that there are stark differences between CEL groups in respect of their constituent numbers of surnames or *f-s* pairs as reported in Table 2.

These differences are a consequence of two processes; a) the high variability in the number of surnames sourced for the full reference list, as indicated in Text S1 (e.g only 80 British, 9 Slavic or 18 Swedish surnames were identified in the literature whereas there are several thousand Turkish, Persian or Arabic surnames), and b) the effect of the operation of matching the reference list with the extracted global dataset (described at the beginning of this section), which results in a selective loss of surnames from CELs for which no records exist in the 17 country global dataset. The selective nature of such missing records might have arisen from historic migration patterns, lack of representativeness in the telephone directories of the countries included here, or data formatting issues beyond our control in terms of transcription and transliteration of names into the Roman alphabet. All of these problems with the reference list suggest the need in future work for a much larger surname reference list that is evenly distributed between CELs. Such an expanded list does not necessarily need to come from published sources, and could potentially be generated synthetically using the current reference list expanded through a family of network classification algorithms known as *label propagation* [8]. We have not attempted this here in order to preserve the complete separation between the independently sourced reference list – acting as the ‘gold standard’ – and the global dataset – our test data. Both of these sources are used for validation purposes, as reported in Tables 3 and 4.

## Discussion

The naming network model proposed here demonstrates the existence of clear cultural naming practices based on much more complex attachments than geographic origins alone, and indicates that socio-cultural practices are sustained for generations after migration. Naming networks thus reveal the links that bind us together in communities of cultural practice, and provide a useful framework for classifying populations into cultural ethnic and linguistic communities.

Our methodology is valuable for detecting the emergence of new naming communities, as well as revealing the ancestral hierarchies of cultural, ethnic, linguistic and religious attachment that underpin existing ones. Sensitivity analysis allows investigation of overlaps and apparent exceptions when defining communities. In the context of millions of individuals across 17 diverse countries, the forcefulness of the evidence presented here is overwhelming.

The patterns that we have identified have been detected independently of geographic location. Extensions of this work might investigate spatial segregation of CEL groups in different societies [37], to monitor minority integration, or analyse how they relate to socioeconomic inequalities, genetic profiles [18], health care needs [41], or ethnic preferences in on-line communities [24]. This research suggests that the net effects of human migration over the last several centuries has been to spawn new ‘naming communities’, and that names remain important pointers to community membership – or the lack of it. Naming practices provide enduring tokens of cultural affiliation in the era of globalisation; conversely, the transience of naming conventions renders them important indicators of population composition over space and the scale and pace of ethnic affinity and cultural change. Inherently vague concepts such as ‘social integration’ of minority groups may be monitored using this approach. A consequence of this work may thus be supplementation of static mapping of fixed cultural and ethnic classifications in national Censuses with a more dynamic understanding of human Diaspora in the broadest sense. We believe that the implications of this for physical, biological and social science research are profound and far-reaching.

## Supporting Information

Text S1 (DOC)
