# Supplementary material for: Ethnicity and Population Structure in Personal Naming Networks
Source: PLoS One. 2011 Sep 6;6(9):e22943. doi: 10.1371/journal.pone.0022943 (PMC3167808; doi:10.1371/journal.pone.0022943)
Supplement: Text S1 — (DOC) [file pone.0022943.s001.doc]

**Supporting Information S1**

Table S1: List of sources used to build the diagnostic surnames list

| **CEL Group** | **Source** | **Peer Reviewed or Nat’l. Stats** | **Number of surnames** | **Reference citation** | **Comments** |
| --- | --- | --- | --- | --- | --- |
| Armenian | Federation of East European Family History Societies | No | 1818 | [1] |  |
| Belgium | Statbel | Yes | 178 | [2] |  |
| British | Mascie-Taylor & Lasker, 1985 | Yes | 85 | [3] |  |
| Cambodian | Tu *et al*, 2002 | Yes | 84 | [4] |  |
| Chinese | Quan *et al*, 2006 | Yes | 1186 | [5] |  |
| Czech | Kysilka, 2009 | No | 99 | [6] |  |
| Danish | Danmarks Statistik (Statistics Denmark) | Yes | 20 | [7] |  |
| Finnish | Finnish Population Registration Centre | Yes | 10 | [8] |  |
| French | Darlu *et al*, 1997 | Yes | 100 | [9] |  |
| German | Kunze, 1999 | No | 57 | [10] |  |
| Greek | Dimitrios, 2009 | No | 405 | [11] |  |
| Hungarian | Hungary’s Ministry of Interior | Yes | 100 | [12] |  |
| Iranian | Yavari *et al*, 2005 | Yes | 25 | [13] |  |
| Irish | Tucker, 2006 | Yes | 13 | [14] |  |
| Italian | Alfemminile.com | No | 148 | [15] | Only the top 150 were taken |
| Jewish | Himmelfarb *et al*, 1983 | Yes | 35 | [16] |  |
| Norwegian | Statistics Norway | Yes | 100 | [17] |  |
| Polish | Poland Ministry of Interior | Yes | 33 | [18] |  |
| Russian | Balanovsky et al 2001 | Yes | 89 | [19] |  |
| Slovenian | Statistical Office of the Republic of Slovenia | Yes | 100 | [20] |  |
| Spanish | Word & Perkins, 1996 | Yes | 890 | [21] | Only the top 890 surnames were taken (heavily Hispanic) |
| **Multi-CEL group** | | | | |  |
| Various Asian | Lauderdale and Kestenbaum, 2000 | Yes | 10925 | [22] |  |
| Various Middle East | Lauderdale & Morrison | Yes | 22362 | [23] |  |
| Polish & Various Slavic | Worldnames | No | 400 | [24] |  |
| Several nationalities | Spanish National Statistics Institute (INE) | Yes | 600 | [25] |  |

# *Notes*: See the reference list at the end of this document for details of each individual citation. The sum total of the number of surnames column is 34,287 but this includes some duplicate entries of surnames that arrived to the reference list from different sources in the Multi-CEL group (the last four rows in the table). The total number of unique surnames is 30,479 as reported in the text.

# References cited in supplementary information
